# Digital versus Conventional Dentures: A Prospective, Randomized Cross-Over Study on Clinical Efficiency and Patient Satisfaction

**DOI:** 10.3390/jcm12020434

**Published:** 2023-01-05

**Authors:** Lana Zupancic Cepic, Reinhard Gruber, Jaryna Eder, Tom Vaskovich, Martina Schmid-Schwap, Michael Kundi

**Affiliations:** 1 Department of Prosthodontics, University Clinic of Dentistry, Medical University of Vienna, 1090 Vienna, Austria; 2 Department of Oral Biology, University Clinic of Dentistry, Medical University of Vienna, 1090 Vienna, Austria; 3 Dental Laboratory, University Clinic of Dentistry Vienna, Medical University of Vienna, 1090 Vienna, Austria; 4 Center for Public Health, Medical University of Vienna, 1090 Vienna, Austria

**Keywords:** conventional dentures, digital dentistry, digital dentures, OHIP-20, patient-related outcomes, prospective clinical study

## Abstract

Digital technology facilitates the manufacturing of complete dentures; however, clinical and patient-reported outcomes are underreported. This prospective, randomized, single-blind cross-over study reports the clinical and patient-related outcomes of 10 edentulous patients receiving digital dentures prepared with the Vita Vionic System and conventional dentures produced from heat-polymerized polymethylmethacrylate resin. Clinical efficiency was stated based on the Sato score for quantitative assessment of complete denture quality. Patient satisfaction was evaluated with the oral health-related quality of life questionnaire (OHIP-20). We report here that the Sato score was slightly higher in patients receiving digital versus conventional dentures with a mean of 73.2 ± 12.3 and 67.4 ± 11.8, respectively (*p* = 0.16). Moreover, upper and lower stability was superior in digital dentures (*p* = 0.03 and *p* = 0.10, respectively), while denture polish was better in conventional dentures (*p* = 0.03). Quality of life was slightly higher in patients receiving conventional compared to digital dentures with an OHIP-20 of 101.7 ± 12.0 and 95.6 ± 24.2, respectively (*p* = 0.33). Taken together and when considering the low power of the study, our findings suggest a trend towards better clinical efficiency of digital compared to conventional dentures, while patient satisfaction remained unaffected by the type of manufacturing.

## 1. Introduction

Oral diseases including dental caries and periodontal disease, as well as dental trauma can lead to tooth loss or even complete edentulism and impair the masticatory function with the risk of developing nutritional problems and other health disorders. Esthetic impairments and psychosocial aspects also have to be taken into account, all of which might ultimately affect patients’ oral-health related quality of life (OHRQoL) [1]. Therefore, oral rehabilitation of tooth loss aims to restore the masticatory efficiency and to improve the quality of life. Full or partial removable dentures represent a common and reliable treatment approach to replace missing teeth and to enable normal speech and mastication [2]. This treatment modality increases the overall oral health-related quality of life and patient satisfaction [3], providing a valuable alternative to the more invasive and expensive implant therapy. There are two main fabrication processes for removable dental prostheses: the conventional and the digital.

Conventional dentures require a complex sequence of clinical and laboratory procedures. First, an accurate preliminary and final impression of the alveolar ridges is taken with border molding and recording of the jaw relationship. Stone casts are mounted in a semi-adjustable articulator with a facebow. Lab technician then chooses different sizes and shapes of prosthetic teeth to create wax dentures to be evaluated by dentist and patient. Upon acceptance or following adjustments, permanent dentures are made [4,5]. There are obviously many fabrication steps for conventional dentures that are time consuming and cost intensive. Thus, there is a demand to shorten the workflow while increasing patient comfort. Over recent years, modified protocols have been proposed to simplify prosthetic procedures [5,6,7,8,9], reducing the number of appointments by combining multiple clinical steps in a single visit and eliminating the aesthetic try-in step. However, fabricating conventional dentures has not been changed in this process.

Digital dentures or digital prostheses arise from the workflow that involves scanning of conventional impressions of edentulous jaws and bite registrations, or previous dentures to obtain data for virtual tooth arrangement and denture base design (computer-aided design—CAD) followed by machine processing (computer-aided manufacturing—CAM) [10]. In addition, intraoral digital impressions can be used to digitize edentulous jaws, however, the accuracy in capturing soft tissue is disputed [11]. Digital records facilitate the reproduction of the denture whenever needed [12]. Digital denture technology uses either additive printing or subtractive milling to create acrylic resin dentures [13]. These dentures were reported to have a more precise base fit and a better retention than conventional heat-polymerized dentures, are less labor-intensive, and can reduce the number of visits required to complete the process to only two appointments [14,15]. Moreover, digital dentures have shown promising short-term clinical performance, positive patient-related results, and reasonable time-cost-effectiveness [16,17,18,19]. However, there is still a limited number of randomized clinical trials to support the superior efficacy of digital dentures concerning patient’s satisfaction and clinical outcomes over conventional prosthesis. 

Patient-reported outcomes are of particular importance when considering optional treatments because they are more sensitive than functional measures in detecting differences between treatments [20]. Consequently, patient satisfaction and oral health-related quality-of-life instruments are commonly used in clinical trials to evaluate prosthetic treatment outcomes [21]. The Oral Health Impact Profile (OHIP) is one of the most popular measures to assess the impact of oral disease on peoples’ quality of life [22]. Increasing evidence suggests that complete dentures manufactured using CAD/CAM result in greater patient’s satisfaction versus conventional methods due to better fit, reduced chair time, shorter appointments, and fewer post-insertion visits [14]. Still, from the perspective of the patients, the fabrication method—digital versus conventional—of the dentures had no significant influence on the quality of life [23]. There is nevertheless a demand to increase the evidence on patient-related outcomes with respect to the digital and conventional prosthesis. Here, we demonstrate the treatment efficacy of a system for designing and milling digital dentures (Vita Vionic Solutions; Vita Zahnfabrik, Bad Säckingen, Germany) in comparison with the conventional method, as measured by clinical and patient-based outcomes. 

## 2. Material and Methods

### 2.1. Study Design and Randomization

**Figure 1:**
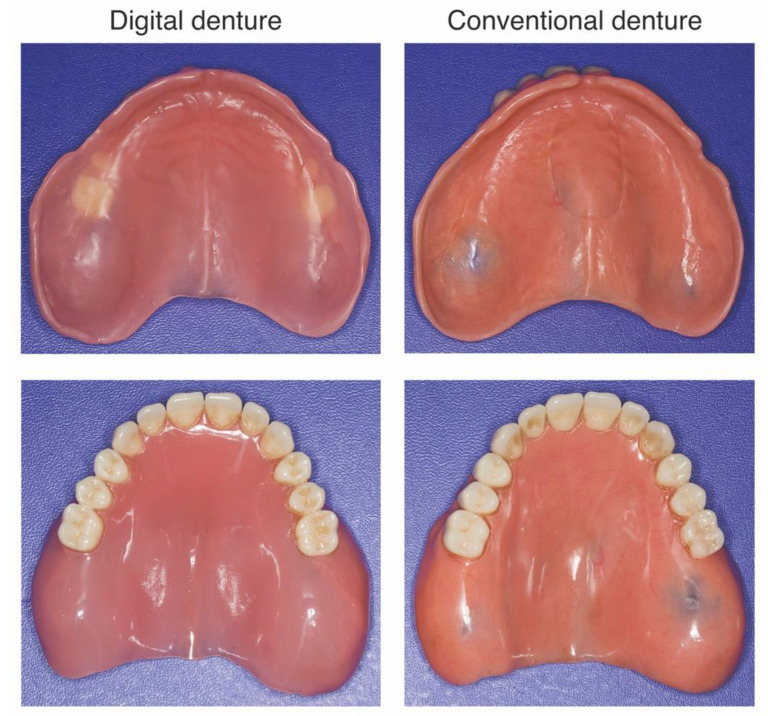
Maxillary digital and conventional denture of the same patient.

This prospective cross-over study was approved by the Ethics Committee of the Medical University of Vienna (EK NR 1062/2018) and patients gave informed and written consent to participate in this study. At total of 10 completely edentulous adult patients, eight men and two women with a mean age of 53.6 years, with alveolar ridges Class II (immediately post extraction), III (well-rounded ridge form, adequate in height and width) or IV (knife-edged ridge form, adequate in height and inadequate in width) according to the classification of Cawood and Howell were enrolled. Exclusion criteria were severely atrophic ridges (Class V and VI), hypertrophic tissue and maxillofacial defects. All patients included required new dentures due to aesthetic or functional impairment after an average 4.9 ± 5.1 years of using their old dentures. Digital and conventional dentures and were randomly assigned to one of the two groups: Group 1 first received the digital dentures and then, after a washout period of one week using the old dentures, the conventional dentures. Group 2 wore the new conventional denture before the washout period and received the digital denture after one week of wearing the old denture. Randomization was accomplished by a computer program. Patients were blinded for the fabrication type of dentures. All treatment was performed by one faculty member (L.Z.C.) of the Department of Prosthodontics with a 10-year experience and by one master dental technician (T.V.). Example of a conventional and a digital denture are shown in Figure 1. 

### 2.2. Conventional and Digital Dentures

Conventional dentures were produced using the compression molding technique with heat-curing acrylics (Promolux C34, Merz Dental GmbH, Lütjenburg, Germany CE 0482) and denture teeth Vitapan Excell for anterior and Vitapan Lingoform for posterior set-up (Vita Zahnfabrik H. Rauter GmbH & Co. KG, Bad Säckingen, Germany CE 0124), which were also available in the tooth library for digital dentures. The gypsum master casts generated through a conventional custom tray impression were first exactly mounted in the articulator (Artex CR, Amann Girrbach AG, Pforzheim, Germany) using the facebow and the maxillomandibular relationship record, then a manual tooth set-up in wax was made for the clinical try-in. Upon their approval, the try-in dentures were transferred to the definitive dentures. In this process, the wax dentures mounted on master casts were embedded in stone in a split flask according to the manufacturer’s instructions. After wax burnout, the acrylic is filled into the gap in a pack-and-press process and polymerized. A lingualized occlusal relationship with front-canine guidance was requested for both types of prosthesis. In contrast to the digital dentures, all conventional maxillary dentures had a posterior palatal seal by erasing the A-line and a relief of the torus palatinus using a tin foil, which are traditionally made to improve the retention properties and wearing comfort of the denture. This resulted in the differences in the tissue surface appearance of the maxillary dentures of the two groups shown in Figure 1. 

**Figure 2:**
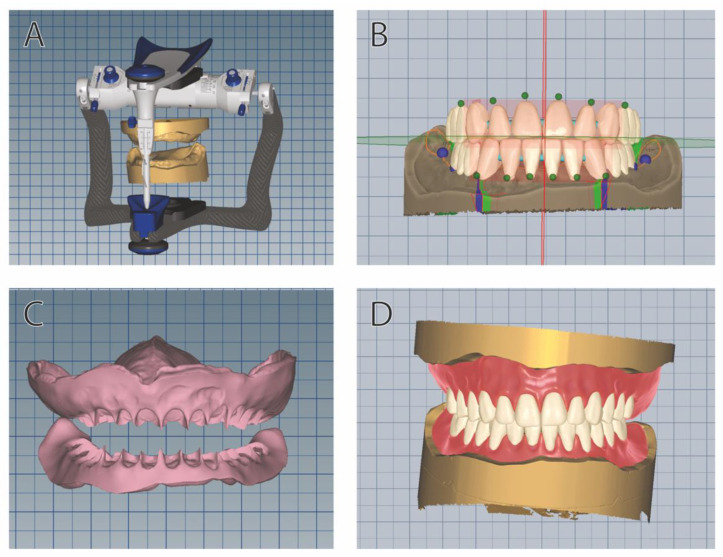
Digital workflow of designing dentures. (**A**) Mounted casts in the virtual articulator. (**B**) Digital tooth arrangement based on software model analysis. Statically correct set-up areas are depicted by lines and planes and fitting tooth sets are automatically positioned in the correct alignment. (**C**) Design of the base. (**D**) Visualization of the final prosthesis prior to its processing.

Digital dentures were created using digital data acquired by scanning (Ceramill MAP 400 Scanner, Amann Girrbach AG, Pforzheim, Germany) the mounted master stone casts mentioned above. This method mainly complies with the approach described by Yilmaz et al. [24] to combine the digital and conventional workflow. Setting up the teeth and customization was performed in virtual articulator using a computer-aided denture design software (Ceramill Mind, Amann Girrbach AG, Pforzheim, Germany). To minimize discrepancies between the analog and digital tooth arrangement, the conventional wax set-up of denture teeth was scanned and used as a reference in the CAD process. For the CAM-processing (Ceramill Motion 2, Amann Girrbach AG, Pforzheim, Germany), the Vita Vionic Solutions (Vita Zahnfabrik H. Rauter GmbH & Co. KG, Bad Säckingen, Germany) material system was chosen. It contains wax and PMMA blanks for the fabrication of wax try-ins and final denture bases, special prosthetic frameworks for the circular and basal CAM-processing of denture teeth, and a bonding agent for the adhesive fixation of teeth in the milled bases. Following successful clinical verification of the digital wax prosthesis (VITA VIONIC WAX), the circular and basal CAM-modification of the VITAPAN EXCELL DD FRAME anterior teeth and the VITAPAN LINGOFORM DD FRAME posterior teeth was performed and the final denture base was milled from a VITA VIONIC BASE PMMA blank. After conditioning of the base and denture teeth, the milled PMMA alveoli were moistened with VITA VIONIC BOND and the teeth were bonded with adhesive. The interdental spaces were closed with veneering composite VITA VM LC flow. After polymerization in the pressure pot, the final polishing was performed. The digital workflow is partially exemplified in Figure 2. 

### 2.3. Clinical Workflow

All clinical steps followed the traditional five-visit complete dentures workflow as follows: preliminary alginate impressions (visit 1); border molding with a modelling compound (Impression Compound, Kerr Corp) followed by final impressions (visit 2) with polysulfide impression material (Permlastic, Kerr Corp, Orange, CA, USA); occlusal registration (visit 3); manual and digital wax teeth try-in (visit 4); delivery according to randomization (visit 5). Any correction needed prior to dentures finalization was entered in the case report, as were any additional clinical visits, including the number of post-insertion adjustment sessions for both fabrication types. A two-week follow-up was performed on each set of new dentures. Finally, the patients stated their preference for one denture type before the respective manufacturing mode was disclosed. The preferred set of dentures was fitted and the second pair of dentures was provided to the patient.

### 2.4. Clinical Evaluation Based on the Sato-Score

Dentures were independently evaluated by two prosthodontists (L.Z.C., J.E.) with at least 5 years of clinical experience using a 3-grade scale (poor = 0, fair = 1, good = 2) to assess clinical parameters describing the quality of dentures: stability under pressure, retention, border extension, finish quality (polish), aesthetics, phonetics, static and dynamic occlusion, and vertical dimension (O.V.D.). Quality items and grading criteria are summarized in Table 1. Interrater agreement was calculated by Cohen’s kappa coefficient [25]. Values were within the range of 0.3 (aesthetics) to 1.0 (upper border extension, lower border extension, phonetics, dynamic occlusion, and vertical dimension) suggesting ‘fair’ to ‘perfect’ agreement between the two examiners. The clinical evaluation grades of seven parameters—lower stability, lower retention, lower border extension, aesthetics, static occlusion, dynamic occlusion and vertical dimension were converted into the Sato score for quantitative assessment of complete denture quality ranging from 0–100 [26].

### 2.5. Oral Health-Related Quality of Life

Patient’s perception of prosthodontic treatment was investigated using the OHIP-20 standardized questionnaire [27] answerable by a six-point Likert scale [28]: never; rarely; occasionally; often; very often; all the time. Each response is assigned a numerical score ranging from one (all the time) to six (never), which were summed up to calculate the total score. The denture related factors (ease of cleaning, general satisfaction with the denture, ability to speak, comfort, aesthetics, stability, and the ability to chew seven index foods—white bread, cheese, beets, sausages, steaks, apples and salad, and general satisfaction with their oral health) were rated by drawing a vertical line on 10-cm visual analogue scales (VAS) ranging from ‘totally dissatisfied’ (0) to ‘completely satisfied’ (10) at the point that best reflected their response [29,30].

### 2.6. Statistical Analysis

The Sato-score was defined as a primary endpoint. A 10% difference was considered clinically relevant. Since the standard deviation of the score was estimated at 12%, this translates into a Cohen’s d_z_ = 1.076, assuming a correlation of 0.7 between scores. To detect such a clinically relevant difference at the 5% level of significance with a power of 80%, a sample of *n* = 9 is necessary. Since a randomized sequence with an equal number of patients was planned, *n* was set to 10. Secondary endpoints were the OHIP score and VAS scale assessments. Only the primary endpoint is relevant for hypothesis testing; *p* values for the other endpoints should be considered exploratory. Interrater agreement was determined using quadratic weighted kappa. Outcomes determined with metric scales were evaluated using analysis of variance and orthogonal linear contrasts comparing old dentures (baseline) with both new dentures and digital versus conventional digital dentures. Normality of residuals was assessed by Kolmogorov–Smirnov tests with Lilliefors’ corrected *p*-values. Box’ M-test was applied to test the symmetry of the variance-covariance matrix. Ordered categorical variables were compared by Wilcoxon signed ranks tests. Statistical evaluations were carried out using Stata 13.1 (StataCorp, College Station, TX, USA). For all statistical tests, *p*-values below 0.05 were considered significant. 

## 3. Results

### 3.1. Clinical Evaluation and Appointments

As a first approach we compared the clinical efficiency of dentures produced by the digital and the conventional technology by implementing the Sato score for quantitative assessment of complete denture quality [26]. Data is shown in Table 2. Both fabrication methods provided dentures with similar clinical efficiency with a Sato score comparable between digital and conventional dentures with a mean of 73.2 ± 12.3 and 67.4 ± 11.8, respectively. In addition, the median values suggest a trend towards a better performance of the digital compared to the conventional dentures with 74.0 (66.0–85.0) and 68.0 (66.0–75.3), respectively. It is particularly the upper and lower stability of the digital dentures that was in favor of the digital compared to the conventional dentures; it is 70% versus 20% optimal upper stability for digital versus conventional dentures (*p* = 0.025). On the other hand, only 20% of the digital dentures exhibited a perfect finish compared to 80% of traditionally manufactured dentures (*p* = 0.034). There is thus a weak cumulative clinical advantage of the digital compared to the conventional dentures highlighting the better stability, while the conventional dentures still have a better finish. 

Several aesthetic corrections were necessary during the try-in session, resulting in one or two additional try-in visits, which resulted in a total number of clinical appointments of 5.3 ± 0.5 and 5.9 ± 1.0 for digital and conventional dentures. The corrections included misaligned occlusal plane, shifted midlines, incorrect incisor edge positions, insufficient lip support, incorrect single teeth axis, and in one participant, the bite registration had to be repeated due to incorrect interocclusal relation. The incidence of denture sore spots differed only marginally between the two types of digital dentures; thus, the average number of post-insertion adjustment sessions was also similar with 0.4 ± 1.3 and 0.3 ± 0.7 for digital and conventional dentures.

### 3.2. Oral Health-Related Quality of Life and Patient Satisfaction

The mean OHIP-20 score was 83.1 ± 27.1 before treatment and is linked to the old dentures. The OHIP-20 score increased to 95.6 ± 24.2 and 101.7 ± 12.0 with digital and conventional dentures, respectively. When considering the medians, OHIP-20 increased from 95.5 (63.0–99.5) before treatment to 105.0 (87.8–110.8) and 106.0 (93.8–109.3) after treatment with digital and conventional dentures, respectively, suggesting an improvement in oral health-related quality of life after receiving new dentures, regardless of the type of fabrication (Table 3). This higher OHIP-20 is mainly due to less Food debris accumulation (0.008), Feeling of uneasiness (p = 0.007) and Inability to enjoy company (0.052). Nevertheless, the OHIP-20 score was comparable between the new digital and conventional dentures (Table 3). In support of the OHIP-20 data, patient satisfaction was higher with the new compared to the old dentures, particularly because of improved Overall chewing efficiency (0.009), and in detail on chewing beets (0.029), sausages (0.022) and apples (0.016). Again, patient satisfaction was independent of the digital and conventional manufacturing of the new dentures (Table 4). 

## 4. Discussion

This study was initiated to increase the evidence for the clinical use of digital prosthesis when compared to the classical conventional prostheses. In this prospective, randomized, single-blind cross-over study, we considered clinical and patient-related outcomes of ten edentulous patients requiring renewal of their old prosthesis. The main finding of the present study was that clinically, digital prosthesis performed better than conventional prosthesis with respect to the upper and lower stability; additionally, the cumulative Sato score for quantitative assessment of denture quality almost reached the level of significance. From a patient’s perspective, there was an improvement in oral health-related quality of life and patient satisfaction after receiving new dentures but independent of the digital or conventional processing. These findings are important because they clearly suggest that renewal of dentures improves the patient’s life quality and satisfaction but not necessarily with digital over conventional dentures. 

If we relate the findings to those of others, we can refer to Peroz et al. who enrolled 16 participants receiving digital and conventional dentures [23]. Consistent with our observations, the median Oral Health Impact Profile, German version (OHIP-G49 score) was similar with the digital and the conventional workflow. As in our study, there are subtle differences in favor of conventional dentures concerning physical pain, functional limitation and feeling of being handicapped [23]. Peroz et al. further reported that the borders of the digital dentures were more frequently overextended at the time of insertion, reducing their retention, especially in the maxilla. Nevertheless, after adaptation, digital dentures and conventional dentures performed similarly [31]. Other reports, however, were clearly in favor of the complete digital dentures with respect to patient’s satisfaction and prosthesis retention compared with conventional dentures [32]. Thus, the accumulating evidence supports the use of digital dentures as the clinical and the patient-reported outcomes are not identical but at least similar to the use of conventional prostheses. 

How can we explain that clinically digital prosthesis performed better than conventional prosthesis with respect to the upper and lower stability? The increased stability of digital dentures could be attributed to differences in processing the acrylic resin for the denture base compared to the conventional technique. Milling dentures from a PMMA puck eliminates the polymerization shrinkage inherent in the conventionally processed PMMA dentures. Polymerization shrinkage requires an accommodation phase to fit conventional dentures and enhance the stability of the milled denture bases [32,33,34,35]. On the other hand, conventional dentures exhibited a faultless finish compared. The integration of the advantages of digital and conventional dentures is presumably the reasons why the cumulative Sato score is almost identical in patients receiving the digital and conventional dentures. Thus, to further improve prosthetic concepts, we have to focus on a detailed analysis and not rely on the cumulative Sato score. 

We additionally have to discuss why, if digital prosthesis performed better than conventional prosthesis with respect to the stability, they have no considerable impact on the oral health-related quality of life and patient satisfaction? It is maybe not surprising that patients notice the advantage of changing the old prosthesis into a new prosthesis; this observation based on OHIP-20 and patient satisfaction data is more of a support of known evidence and does not require extensive discussion—but why are digital and conventional prosthesis similarly appreciated by the patients? Even when considering the details that cumulate in the OHIP-20, there are no obvious differences between digital and conventional prosthesis in the opinion of the patients. Theoretically, we might interpret the findings in favor of digital dentures, which are at least supposed to be manufactured more rapidly and which avoid inconvenient and complex techniques. In reality, however, considering the present study, digital and conventional prosthesis both required an average of five appointments needed to insert the prostheses, thus there is room for improvement of the digital workflow to shorten the number of visits. 

The limitations of this clinical trial include the sample size of only 10 participants, as this was a study of digital dentures in individuals with no severe oral health problems to detect trends and encourage future research. In addition, a longer follow-up to validate the performance of the digital dentures compared to the conventional might be necessary to assess the differences between the tested treatment options. Hence, further research is needed to improve treatment protocols of edentulous patients and to enhance treatment efficiency.

## 5. Conclusions

From the present research, we can conclude that digital and conventional dentures show a similar clinical performance that extends towards the oral health-related quality of life and patient satisfaction. This study can be considered a solid foundation to further refine clinical research in prosthodontics with the overall aim of strengthening the clinical evidence supporting the use of digital dentures in edentulous patients.

## Figures and Tables

**Table 1:** Evaluated clinical parameters of denture quality.

Stability of maxillary/mandibular denture under pressure and functional movements
2: Within tissue displacement of a denture base under rotational/horizontal forces
1: Displacement beyond normal tissue pattern
0: Sliding of a denture base under rotational/horizontal forces
Retention of maxillary/mandibular denture
2: Very good resistance to vertical pulling and lateral force on central incisors
1: Moderate resistance to vertical pulling and little resistance to lateral force on central incisors
0: Poor resistance to vertical pulling and no resistance to lateral force on central incisors
Border extension of maxillary/mandibular denture
2: All satisfactory anatomical points
1: max. three negative findings
0: Overall flange overextension/sub-extension
Denture polish
2: no negative findings
1: one negative finding
0: two or more negative findings
Aesthetics (teeth selection, arrangement of anterior teeth, smile line, lip support)
2: no negative findings
1: one negative finding
0: two or more negative findings
Phonetics
2: proper pronunciation (“S”, “F”, “TH”)
1: discreet lisping and/or mumbling
0: pronounced lisping and/or mumbling
Static occlusion
2: continuously firm intermaxillary contacts in the posterior segment/soft contacts in the anterior segment
1: incorrect intercuspation—up to two premature contacts
0: incorrect intercuspation—three or more contacts to adjust
Dynamic occlusion
2: canine guidance on the working side
1: balance contact in the posterior segment
0: hyper-balanced articulation
Vertical dimension
2: interocclusal rest space 2 to 5 mm
1: interocclusal rest space 1 or 6 mm
0: interocclusal rest space < 1 mm or >6 mm

**Table 2:** Clinical outcomes and Sato-score.

CD Type	Conventional Dentures			Digital Dentures			
Grades	0	1	2	0	1	2	
	*n* (%)	*n* (%)	*n* (%)	*n* (%)	*n* (%)	*n* (%)	*p* Value
Upper stability	0 (0%)	8 (80%)	2 (20%)	0 (0%)	3 (30%)	7 (70%)	0.025
Lower stability ^a^	1 (10%)	8 (80%)	1 (10%)	0 (0%)	6 (60%)	4 (40%)	0.102
Upper retention	0 (0%)	3 (30%)	7 (70%)	0 (0%)	1 (10%)	9 (90%)	0.157
Lower retention ^a^	2 (20%)	6 (60%)	2 (20%)	1 (10%)	7 (70%)	2 (20%)	0.705
Upper border extension	0 (0%)	0 (0%)	10 (100%)	0 (0%)	1 (10%)	9 (90%)	0.317
Lower bd. extension ^a^	0 (0%)	2 (20%)	8 (80%)	0 (0%)	1 (10%)	9 (90%)	0.317
Denture polish	0 (0%)	2 (20%)	8 (80%)	0 (0%)	8 (80%)	2 (20%)	0.034
Aesthetics ^a^	0 (0%)	5 (50%)	5 (50%)	0 (0%)	3 (30%)	7 (70%)	0.414
Phonetics	0 (0%)	0 (0%)	10 (100%)	0 (0%)	2 (20%)	8 (80%)	0.157
Static occlusion ^a^	0 (0%)	9 (90%)	1 (10%)	1 (10%)	6 (60%)	3 (30%)	0.564
Dynamic occlusion ^a^	1 (10%)	4 (40%)	5 (50%)	0 (0%)	5 (50%)	5 (50%)	0.564
Vertical dimension ^a^	0 (0%)	0 (0%)	10 (100%)	0 (0%)	0 (0%)	10 (100%)	1.000
	Mean ± SD	Md (IQR)		Mean ± SD	Md (IQR)		*p* value
Sato-score	67.4 ± 11.8	68.0 (66.0–75.3)		73.2 ± 12.3	74.0 (66.0–85.0)		0.160

CD = complete denture; SD = standard deviation; Md = median; IQR = interquartile range. ^a^ Selected 7 factors used to calculate Sato-Score.

**Table 3:** OHIP-20 evaluation at the baseline (old CD) and after treatment with conventional and digital dentures. A higher OHIP-20 score, reflects a better oral health-related quality of life.

CD Type	OLD (O)		Conventional (C)		Digital (D)		*p* Values	
OHIP-Items	Mean ± SD	Md (IQR)	Mean ± SD	Md (IQR)	Mean ± SD	Md (IQR)	O–D/C	C–D
Chewing difficulties	3.5 ± 1.8	4.0 (1.8–4.8)	4.2 ± 1.5	5.0 (4.0–5.0)	3.7 ± 1.8	4.5 (2.3–5.0)	0.530	0.363
Food debris accumulation	2.6 ± 1.2	2.5 (2.0–3.8)	4.4 ± 1.4	4.5 (4.0–5.0)	4.3 ± 1.3	4.5 (4.0–5.0)	0.008	0.591
Fit of the prosthesis	3.4 ± 1.9	3.5 (1.5–5.0)	4.7 ± 1.5	5.0 (4.3–5.8)	4.1 ± 1.9	4.5 (3.3–5.8)	0.206	0.425
Pain in the mouth	4.4 ± 1.5	5.0 (4.0–5.0)	4.3 ± 1.3	4.0 (4.0–5.0)	4.3 ± 1.9	5.0 (3.0–6.0)	0.879	1.000
Chewing efficiency	3.3 ± 1.7	4.0 (2.0–4.0)	4.5 ± 1.4	5.0 (4.3–5.0)	4.3 ± 1.3	5.0 (3.3–5.0)	0.107	0.678
Wounds in the mouth	4.2 ± 1.8	4.0 (3.3–6.0)	4.2 ± 1.5	4.0 (4.0–5.5)	3.9 ± 1.9	4.0 (3.0–5.8)	0.831	0.604
Discomfort	4.2 ± 1.9	5.0 (3.3–5.8)	5.1 ± 0.9	5.0 (4.3–6.0)	4.6 ± 1.8	5.0 (4.3–6.0)	0.399	0.427
Concerns	4.6 ± 1.9	5.5 (3.5–6.0)	5.5 ± 0.5	5.5 (5.0–6.0)	5.0 ± 1.2	5.0 (5.0–5.8)	0.301	0.138
Feeling of uneasiness	3.7 ± 1.6	4.0 (3.3–4.8)	5.4 ± 0.7	5.5 (5.0–6.0)	5.3 ± 0.9	5.5 (5.0–6.0)	0.007	0.591
Omitting certain foods	3.3 ± 1.6	3.0 (2.0–4.8)	4.4 ± 1.1	4.5 (4.0–5.0)	4.3 ± 1.2	4.5 (4.0–5.0)	0.084	0.678
Impaired dietary habits	4.5 ± 1.7	5.0 (3.3–6.0)	5.0 ± 1.4	5.5 (5.0–6.0)	4.8 ± 1.0	5.0 (4.0–5.8)	0.438	0.662
Inability to eat	4.6 ± 1.6	5.0 (3.5–6.0)	5.4 ± 1.0	6.0 (5.0–6.0)	5.2 ± 0.9	5.5 (4.3–6.0)	0.234	0.509
Interruption of meals	4.5 ± 1.4	4.5 (4.0–5.8)	5.1 ± 0.7	5.0 (5.0–5.8)	4.7 ± 1.7	5.5 (4.0–6.0)	0.405	0.309
Anger	3.8 ± 1.3	4.0 (3.0–5.0)	5.1 ± 0.7	5.0 (5.0–5.8)	4.6 ± 1.6	5.0 (4.0–6.0)	0.054	0.343
Embarrassment	4.5 ± 1.6	5.0 (3.3–6.0)	5.4 ± 0.7	5.5 (5.0–6.0)	5.0 ± 1.5	5.0 (5.0–6.0)	0.163	0.269
Averse to go out	4.8 ± 1.8	6.0 (3.5–6.0)	5.9 ± 0.3	6.0 (6.0–6.0)	5.5 ± 1.6	6.0 (6.0–6.0)	0.121	0.343
Social intolerance	4.9 ± 1.2	5.0 (4.3–6.0)	6.0 ± 0.0	6.0 (6.0–6.0)	5.5 ± 1.3	6.0 (6.0–6.0)	0.053	0.435
Irritability	5.0 ± 1.2	5.0 (5.0–6.0)	5.7 ± 0.5	6.0 (5.3–6.0)	5.4 ± 1.3	6.0 (6.0–6.0)	0.093	0.468
Inability to enjoy company	4.9 ± 1.4	5.0 (5.0–6.0)	5.9 ± 0.3	6.0 (6.0–6.0)	5.6 ± 1.3	6.0 (6.0–6.0)	0.052	0.496
Life less satisfying	4.4 ± 1.8	5.0 (3.5–5.8)	5.5 ± 0.8	6.0 (5.3–6.0)	5.5 ± 1.1	6.0 (6.0–6.0)	0.068	1.000
OHIP-20 TOTAL SCORE	83.1 ± 27.1	95.5 (63.0–99.5)	101.7 ± 12.0	106.0 (93.8–109.3)	95.6 ± 24.2	105.0 (87.8–110.8)	0.116	0.332

*p* ≤ 0.05 represents statistically significant differences in ratings of outcomes between old and both new CD types (O–D/C) and between conventional and digital dentures (C–D).

**Table 4:** Evaluation of patient satisfaction (0–10 visual analogue scale) with the different denture types, including baseline assessment (old dentures). Higher the value, greater the patient satisfaction.

CD Type	Old (O)		Conventional		Digital		*p*-Value	
Satisfaction Factors	Mean ± SD	Md (IQR)	Mean ± SD	Md (IQR)	Mean ± SD	Md (IQR)	O–D/C	C–D
Ease of cleaning	8.9 ± 2.1	9.7 (9.4–9.8)	8.8 ± 1.9	9.6 (9.4–9.9)	9.8 ± 0.2	9.8 (9.7–9.9)	0.631	0.140
Satisfaction with dentures	5.1 ± 3.7	6.1 (2.0–8.1)	8.0 ± 1.9	8.7 (6.3–9.4)	8.3 ± 1.8	9.2 (6.6–9.7)	0.048	0.592
Ability to speak	7.3 ± 3.6	9.6 (4.9–9.8)	9.3 ± 0.5	9.6 (8.8–9.7)	8.6 ± 1.7	9.5 (8.0–9.7)	0.232	0.112
Comfort	5.2 ± 3.8	6.0 (2.0–7.9)	7.8 ± 2.3	8.4 (5.9–9.6)	8.6 ± 1.7	9.5 (7.0–9.8)	0.053	0.294
Aesthetics	6.4 ± 4.0	8.3 (3.9–9.5)	9.4 ± 0.6	9.7 (9.2–9.9)	9.8 ± 0.3	9.9 (9.6–9.9)	0.031	0.121
Stability	4.6 ± 3.8	5.1 (1.2–8.0)	8.0 ± 2.9	9.6 (5.9–9.9)	8.4 ± 2.8	9.8 (8.7–9.9)	0.021	0.300
Overall chewing efficiency	4.4 ± 2.9	4.8 (2.1–5.1)	7.6 ± 1.9	8.4 (6.0–8.8)	7.5 ± 2.7	8.5 (5.7–9.5)	0.009	0.855
Ability to chew white bread	7.8 ± 2.3	8.5 (6.9–9.4)	9.0 ± 1.5	9.5 (8.8–9.9)	8.6 ± 2.4	9.7 (8.9–9.9)	0.277	0.664
Ability to chew cheese	5.0 ± 3.7	4.2 (3.2–8.5)	7.2 ± 3.3	9.0 (4.9–9.8)	7.1 ± 3.3	8.2 (6.4–9.3)	0.067	0.891
Ability to chew beets	3.1 ± 2.7	2.4 (1.4–4.6)	5.9 ± 3.3	7.5 (3.2–8.4)	5.8 ± 3.2	6.9 (3.0–7.8)	0.029	0.786
Ability to chew sausages	3.4 ± 3.1	2.3 (1.5–4.6)	6.0 ± 3.0	7.4 (3.1–8.0)	7.1 ± 2.9	7.9 (6.8–8.9)	0.022	0.122
Ability to chew steaks	4.6 ± 4.1	3.6 (1.1–8.3)	7.1 ± 3.5	8.7 (5.4–9.8)	7.7 ± 3.1	8.9 (7.0–9.7)	0.161	0.524
Ability to chew apples	4.0 ± 3.2	3.2 (1.3–6.4)	7.1 ± 3.0	8.3 (5.6–9.2)	6.8 ± 3.0	7.1 (5.3–9.2)	0.016	0.734
Ability to chew salad	7.0 ± 3.0	7.8 (5.9–9.3)	8.2 ± 2.9	9.4 (8.4–9.8)	8.6 ± 2.0	9.6 (8.3–9.8)	0.175	0.425
Satisfaction with oral health	6.1 ± 3.5	7.3 (4.2–8.8)	7.7 ± 2.8	8.5 (7.4–9.4)	7.7 ± 3.0	9.3 (6.3–9.7)	0.147	0.925
Health affection by oral health status	20.0%		10.0%		10.0%			

*p* ≤ 0.05 represents statistically significant differences in ratings of outcomes between old and both new CD types (O–D/C) and between conventional and digital dentures (C–D).

## Data Availability

Raw data are made available on request.
